# Revisiting the Doctrine of Intertemporal Law

**DOI:** 10.1093/ojls/gqaa058

**Published:** 2020-12-21

**Authors:** Steven Wheatley

**Affiliations:** Professor of International Law, University of Lancaster

**Keywords:** intertemporal law, custom, time, change, A-series, B-series, Chagos Archipelago

## Abstract

There is a tension in the doctrine of intertemporal law outlined by Max Huber in the Island of Palmas case. The first branch demands that the legality of an act be judged by the law in force at the time the act occurs; the second that we take into account any change in the law over time. We see the problem in the 2019 Chagos Archipelago proceedings. The UK argued that the detachment of the Archipelago from Mauritius in 1965 was not unlawful, because it was not regarded as unlawful at the time. The International Court of Justice (ICJ) disagreed, deciding that the detachment was unlawful at that time, but it relied on the 1970 Declaration on Friendly Relations to confirm this conclusion. This article explains why the ICJ’s use of dynamic logic to reach its decision was correct—and what this tells us about the intertemporal doctrine.

## 1. Introduction

The objective of this article is to resolve the tension between the first and second branches of the intertemporal doctrine outlined by Max Huber in the 1928 *Island of Palmas* case. The first branch demands that the legality or validity of an act be evaluated against the standards in force at the time the act occurs. The second requires that we take into account any change in the applicable law over time. The first branch is generally accepted by international lawyers. The second has caused a great deal of controversy and confusion.

We see the ‘intertemporal problem’ in the *Chagos Archipelago* proceedings before the International Court of Justice (ICJ). The UK argued that its detachment of the Chagos Archipelago from Mauritius in 1965, just prior to Mauritian independence in 1968, was not unlawful, because international lawyers would not have recognised the existence of the right of peoples to self-determination *at the time*. The ICJ disagreed, concluding that the self-determination norm crystallised with the adoption of the 1960 General Assembly resolution 1514 (XV), and that its customary status was confirmed with the adoption of the 1970 Declaration on Friendly Relations. The separation of the Chagos Archipelago was, therefore, unlawful *at that time*. There are, though, three difficulties with the Court’s finding: first, no international lawyer would have agreed in 1960 that resolution 1514 (XV) reflected customary international law; secondly, it is difficult to make the case that the ICJ would have reached the same conclusion in the 1960s; and, finally, the lawyers advising the UK government on the legality of the proposed detachment could not see into the future—and the adoption of the Declaration on Friendly Relations.

To make sense of the doctrine of intertemporal law, we have to explain the role of time in the process of change in customary international law, because explicit recourse to the intertemporal rule is only required when the law has changed over time.^1^ In order to do this, we have to be clear *how* we think about the notion of time.^2^ Philosophers have shown that we can either see events moving through time, from the future, through the present and on to the past (this is the ‘A-series’ conception of time); or we can reject the idea that time passes as an objective reality and focus instead on the timeline of events, which can be described in terms of being ‘later than’, ‘earlier than’ or ‘simultaneous with’ each other (this is the ‘B-series’).

Both the A- and B-series conceptions of time come with their own language and logic. For subscribers to the B-series, there is no passage of time, so we must always be able to say that ‘So and so *is* the case’. Tenseless propositions like this do not change over time. B-series thinking is readily apparent in the UK’s pleadings in *Chagos Archipelago*: Because there is no consensus on the existence of a right of peoples to self-determination in the 1960s, there is no violation of international law when Mauritius becomes independent without the Chagos Archipelago. The static logic of the B-series means that this conclusion does not change over time, even when the General Assembly confirms the existence of the self-determination norm with the adoption of the 1970 Declaration.

For followers of the A-series, by way of contrast, the passage of time is real, and everything is observed from the privileged position of ‘now’—the moment which decides whether something is past, present or future. This means that tensed propositions, ie whether something *was*, *is* or *will be* the case, matter. Importantly, tensed propositions can change over time: something that was not the case can change to being something that is the case. The Opinion of the International Court of Justice reflects this kind of dynamic logic: From the privileged position of 2019 (the ICJ’s then ‘now’), the Court was able to determine that the self-determination norm crystallised with the adoption of the 1960 Declaration—even though the ICJ would not have reached this conclusion in the 1960s. The reason for this is straightforward: in 2019, the ICJ was judging the issue with the benefit of hindsight, in the certain knowledge that the General Assembly had confirmed the existence of the self-determination norm in 1970, with the adoption of the Declaration on Friendly Relations.

This article resolves the ‘intertemporal problem’—the problem of deciding which law to apply when the law has changed—by explaining the importance of the passage of time in the identification of rules of customary international law. Section 2 introduces the doctrine of intertemporal law, explaining the way the two branches were formulated in *Island of Palmas*. Section 3 turns to the subject of change in customary international law, because the intertemporal rule is only needed when the law has changed. Section 4 explains why talking about the passage of time is not straightforward, highlighting the differences between the A- and B-series notions of time. Section 5 shows how A- and B-series thinking manifest themselves in discussions about change in customary international law. Section 6 examines the arguments in the *Chagos Archipelago* proceedings and explains why the ICJ was correct to look to the dynamic logic of the A-series. The work concludes by formulating the intertemporal doctrine fully for the first time. The first branch is already explained by Max Huber in *Island of Palmas*. The second contains two parts. The first part confirms that new rules can impose conditions for the continued enjoyment of rights (*change over time*). The second part explains that our conclusions about the applicable law on a given day can change, depending on *when* we examine the available evidence of state practice and *opinio juris* (*change in time*).

## 2. The Doctrine of Intertemporal Law

The doctrine of intertemporal law (literally, ‘between-time law’) is described variously in the literature as the ‘international intertemporal law’, ‘rule of intertemporal law’, ‘intertemporal rule’, ‘intertemporal principle’ and ‘principle of the intertemporal law’.^3^ Whatever the terminology, the doctrine is concerned with what the Institut de Droit International describes as ‘the delimitation of the temporal sphere of application of norms’^4^ and Judge Mohamed Bennouna calls ‘the application of international law in time’.^5^ The intertemporal rule, then, is a secondary, and not a substantive, rule: it tells us which law to apply to the facts.^6^

Whilst the intertemporal rule is known to every international lawyer,^7^ agreement on its exact content has proved to be somewhat elusive.^8^ This is, in large part, because of its formulation in the 1928 *Island of Palmas* award.^9^ Even a sympathetic reading concedes that this has ‘generated not a little confusion and led to a lively controversy’.^10^ The case involved a dispute concerning sovereignty over the Island of Palmas (or Miangas). The United States’s claim was based primarily on the discovery of the island by Spain in the 16th century and the subsequent transfer of the Spanish title to America under the 1898 Treaty of Paris. The Dutch argument was based on its effective occupation of the island from the 18th century onwards. Both parties were agreed on the timeline of events. They also agreed that

‘a juridical fact must be appreciated in the light of the law contemporary with it, and not of the law in force at the time when a dispute in regard to it arises or falls to be settled’.^11^

This is the first branch of the intertemporal doctrine.

The arbitrator, Max Huber, also accepted the Dutch argument that the change in the law on the acquisition of title to colonised territories, from around the 18th century, requiring that acquisition must be effective, could not be ignored. Consequently, whilst conceding that discovery could give absolute title in the 16th century, according to the applicable law at that time,^12^ he concludes that ‘discovery alone, without any subsequent act, cannot *at the present time* suffice to prove sovereignty over the Island of Palmas’.^13^ Huber explains his reasoning in the following terms:

As regards the question which of different legal systems prevailing at successive periods is to be applied in a particular case (the so-called intertemporal law), a distinction must be made between the creation of rights and the existence of rights. The same principle which subjects the act creative of a right to the law in force at the time the right arises, demands that the existence of the right, in other words its continued manifestation, shall follow the conditions required by the evolution of law.^14^

This is the second branch of Huber’s doctrine of intertemporal law.

Whilst the first branch is generally accepted by international lawyers, the second has aroused a good deal of controversy.^15^ There are at least four different ways the second branch has been explained. First, that its inclusion was a mistake,^16^ with Sir Hersch Lauterpacht claiming that its addition to the intertemporal doctrine represented ‘a clear departure from the views expressed on this subject by a number of international lawyers’.^17^ Secondly, that Huber never intended the distinction he makes between the creation and the continued existence of rights to be applied outside of the law on territory. This is Dame Rosalyn Higgins’s understanding.^18^ Thirdly, that the intertemporal doctrine resolves the conflict between the old and the new rule by destroying the old. This is Philip Jessup’s reading, based on the fact that there was no discussion of the abandonment of the Island of Palmas by Spain ‘because the original sovereignty [acquired by discovery alone] … ceased to exist by virtue of the principle of “intertemporal law”’.^19^ Finally, there is Huber’s own understanding, that the intertemporal rule is needed to resolve the conflict between the law of the 16th century and that of the 18th century onwards, to explain which legal system is to be applied.^20^ But if, as Huber assumes, Spain acquired title to the Island by way of discovery in the 16th century, he must explain how Spain lost that title, or why the Netherlands had a better claim by way of effective occupation,^21^ and he does neither. Daniel-Erasmus Khan concludes: ‘Most likely, the only way out is to frankly admit that the gist of what Huber writes here lies somewhere else … with his theoretical conviction that international lawyers should strive for a dynamic understanding of international law.’^22^

Since the publication of the *Island of Palmas* award, little has been written on the intertemporal doctrine, which applies to all rules of customary international law.^23^ English language textbooks on public international law deal with the issue in the chapter on territory, often simply repeating Huber’s formula for the first branch^24^ and noting the ‘highly controversial’ nature of the second.^25^ Ulf Linderfalk concludes that the ‘sharp reactions’ to the application of the second branch in *Island of Palmas* ‘seem to have made scholars hesitant to approach it’. He gives the example of the Institut de Droit International’s 1975 resolution on ‘The Intertemporal Problem’, which discussed the first branch in detail, but said ‘practically nothing about the validity and significance of the second’.^26^ The only (implicit) reference to the second branch can be found in the preamble, which observes that ‘any solution of an intertemporal problem in the international field must take account of the dual requirement of development and stability’.^27^

## 3. Change in Customary International Law Over Time

The focus here is the application of the intertemporal doctrine in customary international law.^28^ The contemporary formulation of the idea that the law of nations can be identified by examining the customary practices of states is found in article 38(1)(b) of the Statute of the International Court of Justice, which lists as one of the sources of international law ‘international custom, as evidence of a general practice accepted as law’. Whilst the provision is badly drafted, there is agreement that it outlines a two-element approach: to show the existence of custom, there must be: (i) evidence of a general practice; and (ii) evidence of a belief that the practice is required by international law (the *opinio juris* element). In its 2019 *Chagos Archipelago* Opinion, the ICJ observed that the elements of state practice and *opinio juris*, ‘which are constitutive of international custom, are closely linked’. The Court then repeated its established *North Sea Continental Shelf* formulation for the identification of custom:

Not only must the acts concerned amount to a settled practice, but they must also be such, or be carried out in such a way, as to be evidence of a belief that this practice is rendered obligatory by the existence of a rule of law requiring it.^29^

Whilst the complexities associated with the identification of custom have again become the focus of scholarly concern, the subject of time has not featured in the debates. The renewed interest follows the adoption by the International Law Commission (ILC) of a set of draft Conclusions on the identification of customary international law.^30^ The special rapporteur, Sir Michael Wood, had originally identified the ‘time element’ as one of the central issues to be examined—along with state practice and *opinio juris*^31^—but the draft Conclusions do not deal directly with the subject of time, with Sir Michael explaining that their aim is to assist in the identification of customary rules ‘as of a particular time’, not to explain the influences and processes involved in their development ‘over time’.^32^

There are, though, at least three ways that time must feature in any discussion of custom. First, time is an important element in the creation of customary international law, because evidence of state practice and *opinio juris* only emerges over time.^33^ There is an implied recognition of this in ILC draft Conclusion 8(2), which establishes the following: ‘Provided that the practice is general, no particular duration is required.’^34^ The Commentary refers to this as ‘the time element’ in custom.^35^ Here, the International Law Commission follows the well-known dictum in *North Sea Continental Shelf*, where the International Court of Justice accepted that, whilst the passage of only a short period of time is not a bar to the emergence of a new rule, ‘within the period [of time] in question’, there must be sufficient evidence to show the existence of a general practice accepted as law.^36^ The amount of time required is relative:^37^ it all depends on the time it takes for the necessary evidence to emerge.^38^

Secondly, we need the element of time to explain the possibility of change, because custom cannot say incompatible things on the same subject at the same time. The determination of the existence, scope and content of a customary norm (CIL N) requires evidence of a general practice (G/P) *plus* evidence of a belief that the practice is required by international law—the *opinio juris* element (O/J). Thus, in simple terms, G/P + O/J = CIL N.^39^ The same test applies for the identification of any change in the law.^40^ Thus, we see a change from the old rule (CIL N1) to the new (CIL N2) when we see a change in the available evidence of state practice and/or *opinio juris*. The formulation G/P + O/J = CIL N1 changes to G/P + O/J = CIL N2. Both of these propositions are true. CIL N1 is evidenced by a general practice accepted as law. The same is true of CIL N2, even though CIL N1 and CIL N2 say different things about the same subject. But they do not say contradictory things at the same moment. We need, then, the element of time to explain the possibility of change in customary international law.

Finally, we must, as Judge Cançado Trindade pointed out in *Request for Interpretation* (albeit without further explication), ‘be aware of the influence of *the passage of time* … in the evolution of the rules of international law’.^41^ The problem, as the following section makes clear, is that thinking about the passage of time is not straightforward. But, as it turns out, working out how we think about time is the key to understanding the applicable law on any given day – and, consequently, resolving the intertemporal problem.

## 4. Thinking About the Passage of Time

Whether we think in terms of calendar time, and the coming and going of the academic year, with the changing of the seasons,^42^ or clock time, and the constant ticking of our watch,^43^ most of us feel that the passage of time must be an objective feature of reality, and not just a projection of the human mind. The author HG Wells explains the point this way:

You know of course that a mathematical line … has no real existence … Neither has a mathematical plane. These things are mere abstractions … Nor, having only length, breadth, and thickness, can a cube [that does not last for any time] have a real existence … ‘Clearly,’ the Time Traveller proceeded, ‘any real body must have extension in four directions: it must have Length, Breadth, Thickness, and–Duration.’^44^

It was the scientist Sir Isaac Newton who first argued that everything existed in time and space, that time had a separate reality and that the flow of time did not depend on change. In making the case, Newton distinguished between ‘Relative, apparent, and common time … for example, an hour, a day, a month, a year’, and what he called ‘Absolute, true, and mathematical time, [which] *flows uniformly* and by another name is called duration’.^45^

Today, scientists do not accept the Newtonian view of absolute time, or that time flows smoothly at the same rate everywhere in the universe. Following the insights of Albert Einstein on the subject of relativistic space-time,^46^ all time is seen as local time, with a patchwork of local times each measured by observers moving relative to each other.^47^ Yet most of us still follow Newton in thinking that time flows.^48^ This is reflected in our metaphors about time: we speak about events moving through time—from the future, through the present and on to the past, just like sticks float downstream—towards us, past us and then beyond.^49^ But there is a way that we can describe events which does not require the element of time: we can simply arrange them in relation to each other, saying that one event is ‘later than’ or ‘earlier than’ another. Instead of thinking about events flowing through time, we can think about time as a series of events. The philosopher PJ Zwart explains the argument this way: ‘Events do not just have their places in time, like pieces of wood floating in a river, but events constitute time.’^50^

### A. The A- and B-Series

The contrasting ways we can think about time were first outlined in a 1908 article by the metaphysician, John McTaggart. The first possibility is that an event can be referred to in dynamic terms, as moving through time, ‘from the far past through the near past to the present, and then from the present to the near future and the far future’. This McTaggart calls the ‘A-series’ conception of time. Paradoxically, the same event can also be described in static terms—of being in a permanently fixed relationship with other events, being ‘Earlier than some, and Later than some’. This is the ‘B-series’.^51^ We can, for example, say both that the assassination of President John F Kennedy was once future, then present, but is now past (‘A-series’) and that Kennedy died ‘later than’ his election (‘B-series’). McTaggart maintains that the B-series does not work because a proper account of time requires change, and the relationships between events in the B-series do not change—Kennedy always dies after his election.^52^ So this leaves the A-series, the idea that all events must have the intrinsic properties of being future, present and past. But, he argues, it is inconsistent, and therefore illogical, to say that ‘Each event [like JFK’s assassination] is *in the same moment* in the future, in the present, and in the past’.^53^

Whilst McTaggart’s argument for ‘the unreality of time’ has not been generally accepted, his division between the dynamic A-series and static B-series has provided the starting point for most philosophical discussions about time in the past 100 years. For the A-series theorist, the passage of time is real,^54^ and events, such as Kennedy’s assassination, are always first future, then present, then past—never the reverse. For the B-theorist, on the other hand, there is no passage of time: time is just a way of explaining the relationships between events,^55^ which can be arranged in terms of being ‘earlier than’, ‘later than’ or ‘simultaneous with’ other events.^56^

The A- and B-series notions of time each come with their own language and logic, with the A-series providing a tensed theory of time and the B-series a tenseless theory.^57^

For the A-series theorist, the passage of time is real, and our use of tense reflects the observer-independent reality of something being future, present *or* past.^58^ In other words, it really does matter whether we say that something *will be*, *is* or *was* the case. So, for example, after his election on 8 November 1960 and before his inauguration on 20 January 1961, people could say that ‘JFK *will be* President’; between 20 January 1961 and 22 November 1963, they could say that ‘JFK *is* President’; and after 22 November 1963, that ‘JFK *was* President.’ The key point is that these tensed statements are only true at these times.

For the B-series theorist, the passage of time is an illusion, and events can only be described in terms of their relationship with each other.^59^ So the B-theorist would simply say that ‘20 January 1961 *is* the day of JFK’s inauguration’, that ‘his inauguration *is* later than his election’ and that ‘his inauguration *is* earlier than his assassination’. This timeline of events does not change, and it does not matter when these tenseless statements are uttered.

A focus on tense has important implications for the way we frame our truth claims. There is, for example, a difference between saying that ‘2 + 2 *is* 4’, which is true for all time, and saying that ‘It *is* raining’ (what we mean is that ‘It *is* [now] raining’).^60^ For the B-series theorist, there is no passage of time, so any proposition must be true for all time: we must always be able to say that ‘So and so *is* the case’. For the A-series theorist, any proposition must include the tensed property of pastness, presentness or futurity: we must be clear whether something *was*, *is* or *will be* the case. For the A-theorist, as the philosophers John Carroll and Ned Markosian point out, ‘propositions have truth values *at times*, and can change their truth values over time’.^61^ This difference in the ways we can frame our truth claims turns out to be central to the problems that international lawyers encounter when trying to explain change.

## 5. A-Series and B-Series Thinking in International Law Scholarship

The previous section showed that we can either think about time in terms of events moving from the future, through the present and on to the past (A-series) or in terms of the relationships between events (B-series). The key distinction is that the A-series demands the use of tensed language and logic, whilst the B-series requires tenseless language and logic. We see the importance of this difference in the ways that international lawyers speak about change in customary international law over time.

### A. International Law on a Given Day

The intertemporal problem, that is, the difficulty of identifying the applicable law on a given day, arises when there has been a change in the law. We know that custom changes because the law says different things on the same subject, albeit not at the same time. Whilst the possibility of change is not disputed,^62^ there are two well-known paradoxes that emerge when we try to explain the process. The paradox of change results from the fact the early adopters of any new practice must violate the existing law, in order to change the rule by changing the general practice.^63^ This leads to what Sir Gerald Fitzmaurice calls ‘the most striking and difficult paradox’ of international law relying on ‘rule-breaking practice for a new rule of customary international law to come into existence’.^64^ The chronological paradox results from the need to provide evidence that the practice is ‘accepted as law’.^65^ The problem is that those states looking to rely on a new rule cannot genuinely believe the rule already exists. The consequence, as Michael Byers points out, is that there can only be *opinio juris* in respect of those rules ‘*already* in force’,^66^ thus preventing the emergence of any new rules.^67^

The best-known attempt to wrestle with the problems of change in customary international law is found in James Crawford’s 2002 article on ‘International Law on a Given Day’ (written with Thomas Viles). Here, Crawford asks us to consider the content of the international law system on 29 September 1945. This is the day after the Truman Proclamation, by which the United States claimed the exclusive right to exploit its continental shelf, following the discovery of oil and gas. On the day before the Proclamation, the legal position is clear: a state cannot claim exclusive rights to ‘its’ shelf, thereby limiting the rights of other states. On 29 September, the United States is the only state making such a claim, so the Proclamation is not consistent with the existing law. Nor does the Proclamation have ‘the immediate effect of a piece of legislation’. But we also know that the law changes, at some point, from CIL N1 (the coastal state does not have the exclusive right to exploit its shelf) to CIL N2 (the coastal state does have such a right). It would, consequently, as Crawford points out, be odd to treat the Truman Proclamation, which articulated a principle that came to be widely accepted, ‘as unlawful at the time’. He then asks: ‘If it was unlawful, at what point did it become lawful? We seem to have … a question that cannot be answered, of conduct that was neither lawful nor unlawful (or perhaps contingently both).’^68^

The problem that Crawford identifies in relation to the validity of the Truman Proclamation results from B-series thinking. Recall that, for the B-theorist, there is no flow of time: all we can talk about are events, with each event being ‘earlier than’, ‘later than’ or ‘simultaneous with’ other events. The Truman Proclamation is one event. So is any iteration of a customary rule that endures for a certain period.^69^ According to B-series thinking, the job of the law applier, when the law has changed from the old rule (CIL N1) to the new (CIL N2), is to determine which iteration is ‘simultaneous with’ the relevant facts. The reasoning in this case proceeds as follows: (1) 28 September 1945 *is* the date when the United States adopts the Truman Proclamation (note the tenseless use of language); (2) CIL N1 *is* the applicable law on the given day, because the Truman Proclamation cannot, by itself, change the law to CIL N2; (3) because the Truman Proclamation is ‘simultaneous with’ CIL N1, the claim made by the United States *is* invalid, and the United States does not, therefore, have the exclusive right to exploit its continental shelf;^70^ (4) the tenseless logic of the B-series means that this conclusion does not change over time, notwithstanding the subsequent change in the law in this area. Herein lies the basis for the paradox of change: the Truman Proclamation, which led to a change in the law, remains an invalid act.^71^

### B. Custom’s Future, Present and Past

Crawford returns to the subject in his 2013 Hague Lectures, arguing that we should abandon the idea we could write a textbook detailing all of the valid rules of international law on any given day.^72^ He concludes: ‘We simply cannot tell what the law is on a specific day except after the fact.’^73^ This is, in large part, because of the problem of identifying the moment when the law changes from the old rule to the new. The International Law Commission’s special rapporteur on custom, Sir Michael Wood, maintains that this is, in fact, an impossible task, because ‘the creation of customary international law is not an event that occurs at a particular moment’,^74^ but rather (quoting Crawford) ‘emanates from an “intensive dialectic process” between different actors of the international society’.^75^ Reflecting on the ILC draft Conclusions on the identification of custom, Sir Michael explains that their aim is ‘to provide guidance as to whether, at a given moment, it may be said that such process *had occurred*’.^76^ He concludes: ‘Much depends upon the point in time at which evidence is considered.’^77^

We see the importance of the time we examine the available evidence of state practice and *opinio juris* in relation to the UK’s claim to its continental shelf, made in 1964.^78^ The 1963 edition of *Brierly’s Law of Nations*, written by Sir Humphrey Waldock, observes that the law in this area ‘has undergone a radical change … set in motion by President Truman’s Proclamation’, but the textbook does not confirm the existence of a customary rule.^79^ Waldock must conclude, therefore, that the UK’s claim is invalid, and this was the position of most international lawyers at the time.^80^ Yet, the 2019 edition of *Brownlie’s Principles of Public International Law*, written by James Crawford, argues that article 2 of the 1958 Geneva Convention on the Continental Shelf, which recognised the exclusive rights of the coastal state, reflected customary law at the time of its adoption.^81^ For Crawford, then, the UK’s claim must be valid at that time. But now we have to explain how we can have two different answers as to the law applicable in 1964.

The intertemporal problem is the difficulty of deciding which law to apply when the law has changed. There is no issue with international lawyers looking to the B-series to outline the timeline of facts with juridical relevance. This does not change over time. The trouble comes when they rely on B-series logic to determine the applicable law, because of the need for tenseless language, meaning that their conclusions cannot change over time, even when the law changes. That leaves the A-series, based on an understanding that the passage of time is real. We must, then, think in terms of custom evolving from the future, through the present and on to the past; recognise that we identify customary norms from the privileged position of ‘now’; be clear whether our claims about custom ‘were’, ‘are’ or ‘will be’ true;^82^ and, finally, accept the possibility that our conclusions about custom can change over time. We see the implications of this kind of A-series logic in the ICJ’s *Chagos Archipelago* Opinion.

## 6. *The* Chagos Archipelago *Opinion*

Although the intertemporal rule is formally involved in every case before the International Court of Justice, including where the Court applies present-day law to present-day facts,^83^ there are some cases when the ICJ must be explicit about which law is to be applied. We see this in its 1975 *Western Sahara* Opinion, where the Court confirmed that the validity of the acquisition of the territory was to be determined by reference to the law in force ‘at the time’ of its colonisation by Spain.^84^ The International Court of Justice has never, though, clearly set out its understanding of the intertemporal doctrine.^85^ Its 2019 Opinion on *Legal Consequences of the Separation of the Chagos Archipelago from Mauritius in 1965* represented, therefore, a major opportunity for the ICJ to clarify the rules, because the outcome depended, in large part, on *when* the right to self-determination crystallised as a customary norm.^86^ Whilst the Opinion does not deal directly with the issue (neither the term ‘intertemporal law’ nor any of its synonyms are mentioned), the Court’s rejection of the UK’s legal reasoning and its own analysis of the issues allow us to draw some important conclusions about the intertemporal doctrine.

### A. The Arguments

All participants in the *Chagos Archipelago* proceedings were agreed on the timeline of events. The first colonial administration of Mauritius was established in 1715 by France and the territory was ceded to Great Britain by the 1814 Treaty of Paris. Between 1814 and 1965, the Chagos Islands were administered by the UK as a dependency of the colony of Mauritius. On 8 November 1965, the UK established the British Indian Ocean Territory, detaching the Chagos Archipelago from Mauritius. On 12 March 1968, Mauritius became an independent state—without the Chagos Islands.

The participants were also agreed that the right of peoples to self-determination is now a rule of customary international law. The difference in the legal arguments lay in the timing of the crystallisation of the self-determination norm and its status in the period 1965 to 1968.

#### (i) The argument of the UK

The position of the UK was that its detachment of the Chagos Islands from Mauritius was not unlawful, because ‘a legal right to self-determination did not emerge until *after* the 1960s’.^87^ The UK’s written memorandum explains the point this way: ‘According to the inter-temporal rule, the applicable law … is the law of the relevant time’. Consequently, the ICJ should apply the customary law ‘as it stood at the relevant time, and not in the light of how the law subsequently developed’.^88^ In other words, the ICJ should decide the case in the same way it would have done in the late 1960s, a time when the legal status of the self-determination norm divided states,^89^ the ICJ refused to examine the legality of South Africa’s extension of the policy of *Apartheid* to the trust territory of South West Africa,^90^ and international lawyers, with the notable, but solitary, exception of Rosalyn Higgins,^91^ did not accept the customary status of the right of peoples to self-determination.^92^ Had, then, the question of the Chagos Archipelago been put to the ICJ *at the time*, the UK could be confident that the Court would have found its actions to be lawful.

#### (ii) The Opinion of the International Court of Justice

The International Court of Justice, by way of contrast, concluded that a right of peoples to self-determination crystallised on 14 December 1960, with the adoption of General Assembly resolution 1514 (XV). According to the Court, this represented ‘a defining moment in the consolidation of State practice on decolonization’.^93^ The ICJ explained that, ‘although resolution 1514 (XV) is formally a recommendation, it has a declaratory character with regard to the right to self-determination as a customary norm’.^94^ The Court then noted the adoption of the 1970 Declaration on Friendly Relations, which ‘confirmed its normative character under customary international law’.^95^ Because the self-determination norm applied to the whole of the non-self-governing territory, the UK’s detachment of the Chagos Archipelago from Mauritius was unlawful *at that time*.^96^

### B. Explaining the Difference

How do we explain the difference in the legal arguments of the UK and the International Court of Justice on the legality of the detachment of the Chagos Archipelago? Did the UK make a mistake when it claimed the Court should decide the case in the same way it would have judged the issue in the 1960s? Did the Court retrospectively invalidate the lawfulness of the detachment of the Islands in 1965? Did the ICJ get the law wrong when it looked at the 1970 Declaration to confirm its understanding of the law applicable in the period 1965–68? Or did the Court agree with Cyprus that the second branch of the intertemporal rule, outlined in the *Island of Palmas* case, requires that the self-determination rule ‘as it stands today’ should be applied to the events of the past?^97^

There is no disagreement between the UK and the International Court of Justice on the application of the first branch of the intertemporal doctrine: the legality of the detachment of the Chagos Islands was to be judged against the rules in force at the time the act occurred.^98^ The difference resulted, then, from conflicting understandings of the second branch of the intertemporal doctrine and the implications of the change in the law on self-determination for the law applicable in the period 1965–68. The claim here is that we can only make sense of, and explain, this disparity if we take into account the contrasting ways the UK and the ICJ impliedly understood the function of time in the identification of the customary self-determination norm.

#### *(i) A-series & B-series thinking in the* Chagos Archipelago *proceedings*

B-series thinking is readily apparent in the UK’s submissions to the Court. Recall that, for the B-series theorist, there is no passage of time: all that matters are events, which can be organised in terms of being ‘earlier than’ or ‘later than’ each other. The UK’s argument can be summarised as follows: (i) there is no consensus on the customary status of the self-determination norm on 14 December 1960, the date of the adoption of Resolution 1514;^99^ (ii) the General Assembly adopts the Friendly Relations Declaration on 24 October 1970, ‘the first consensus resolution on the right of self-determination, with the United Kingdom joining the consensus’;^100^ (iii) because the detachment of the Chagos Archipelago in 1965 occurs ‘later than’ the adoption of the 1960 Resolution but ‘earlier than’ the 1970 Declaration, there is no violation of international law at this time; and (iv) the tenseless logic of the B-series means that this conclusion must remain true for all time: it does not change even when a legal right to self-determination emerges ‘after the 1960s’.^101^

The Opinion of the International Court of Justice, by way of contrast, reflects the dynamic logic of A-series thinking. Recall that, for the A-series theorist, the passage of time is real, and everything is observed from the privileged position of ‘now’. The Opinion begins by explaining that the Court must determine, ‘first, the relevant period of time for the purpose of identifying the applicable rules of international law and, secondly, the content of that law’.^102^ But the Court goes on to say that, whilst its determination of the applicable law must focus on the period from 1965 to 1968,

this will not prevent it, particularly when customary rules are at issue, from considering the evolution of the law on self-determination since the adoption of the [1960] resolution 1514 (XV) … Indeed, State practice and *opinio juris* … are consolidated and confirmed gradually over time.^103^

The International Court of Justice further decided that it ‘may also rely on legal instruments which *postdate the period in question*, when those instruments confirm or interpret pre-existing rules or principles’.^104^ The Court then refers to the 1970 Declaration on Friendly Relations, explaining that, ‘By recognizing the right to self-determination as one of the “basic principles of international law”, the Declaration confirmed its normative character under customary international law’.^105^ There are three points to note here. First, the ICJ can only look at materials that postdate the period in question from the privileged position of ‘now’ because, during the period in question, international lawyers could not have known about documents to be adopted in the future. Secondly, there is no point looking at later instruments unless there is some new understanding to be gained, either to confirm the existence of a rule or interpret its meaning. Finally, reference to ‘pre-existing rules or principles’ confirms that, as a general rule, the doctrine of intertemporal law prohibits the retrospective application of international law norms.^106^

### C. *Why the ICJ Was Correct in Its Approach*

The UK reacted to the ICJ’s conclusion, in *Chagos Archipelago*, that the country was responsible for an unlawful act of a continuing character, by noting that ‘An Advisory Opinion is advice[;] it is not a legally binding judgment [and] we do not share the Court’s approach’.^107^ The UK is correct. Advisory opinions are just that: ‘advisory’,^108^ and without ‘binding force’.^109^ But this does not mean they are without persuasive authority,^110^ and the ICJ has described their function in terms of providing ‘authoritative legal guidance’.^111^ The UK clearly believed it had a strong case, focused on the first branch of the intertemporal doctrine, which demanded that the Court evaluate the legality of its actions by the standards in force in the 1960s. The fact that the ICJ relied on the 1970 Friendly Relations Declaration therefore required some explanation—which the Court did not give. The UK might, then, argue that the International Court of Justice had fallen ‘into the error of judging the events of long ago by present day standards’.^112^

But there are good reasons to consider that the International Court of Justice did not make a mistake, if we see its reliance on the 1970 Declaration to confirm the applicable law in the 1960s in light of A-series thinking about time. Looking back from the privileged position of 2019 (the ICJ’s then ‘now’), the Court could see the consolidation of evidence for the existence of a general practice accepted as law, including materials that post-dated the detachment of the Chagos Archipelago in 1965 and the independence of Mauritius in 1968. It was then able to determine that the self-determination norm crystallised with the adoption of the 1960 Declaration, even though the Court would not have come to this conclusion at the time. Moreover, the tensed logic of the A-series tells us that two apparently conflicting propositions can be true: that the International Court of Justice would not have recognised a customary right of peoples to self-determination in the late 1960s, and would have found the detachment of the Chagos Archipelago to be lawful *at the time*; and that the ICJ could, with the benefit of hindsight, identify the 1960 Declaration as the defining moment in the crystallisation of the self-determination norm, and therefore conclude that the detachment was unlawful *at that time*.

The first reason to accept that the International Court of Justice was correct in its application of A-series thinking—based on an understanding that the passage of time is real—is that international lawyers, like most people,^113^ believe that the passage of time is real, whatever the scientific reality.^114^ The point is significant because the existence of international law is not a ‘brute fact’ of the world, like the existence of mountains.^115^ International law exists because states and international lawyers think that it exists, and then act as if it exists. Thomas Schultz explains the point this way: ‘Thinking about international law effectively creates international law—not in the sense of wishful thinking, but in the sense of labelling, recognising, characterising.’^116^ Part of that thinking involves a belief in the passage of time as one part of the social construction of international law by international lawyers.^117^ We see numerous examples of this in our legal reasoning, from the influence of past precedents on present-day arguments to the difference we draw between the *lex lata* (the law that presently exists) and the *lex ferenda* (the law of the future).^118^ For international lawyers, time is the temporal measure that tracks the progression of the international law system from the future, through the present and on to the past.^119^

The second reason to be convinced the ICJ was correct in its reliance on A-series thinking, based on a belief that the passage of time is real, is that the Nobel prize winning chemist^120^ Ilya Prigogine has shown there is an arrow of time in complex systems,^121^ like customary international law.^122^ In the same way that a hot cup of coffee always cools (and never heats spontaneously), complex systems always move from the future, through the present and on to the past (never the reverse).^123^ The notion of a complex system is applied where the system emerges from the actions of its component elements; evolves through changes in the behaviours of those elements; and, in turn, influences the component elements’ actions.^124^ The idea is readily applied to customary international law, which emerges from the physical and verbal acts of states,^125^ in the form of state practice and *opinio juris*;^126^ evolves as the result of changes in states’ behaviours,^127^ as they respond to the actions of other states and events in the world;^128^ and constrains the very same states that brought the customary rules into existence in the first place.^129^

Prigogine explains that the arrow of time in complex systems—which always move from the future, through the present, and on to the past—is a consequence of the fact the system evolves as the result of contingent decisions taken by component agents.^130^ This means that there is no guarantee that, if we could rewind and replay the story of the system’s evolution, the same actors would take the same decisions again, in the same circumstances.^131^ We see this in the evolution of the law on self-determination, from President Woodrow Wilson’s call for an independent state for the Polish people in his 1918 address to Congress; to the decision to establish the League of Nations Mandate scheme at the 1919 Paris Peace Conference; to the recognition of the special status of colonised territories in the United Nations Charter; to the adoption of the 1960 General Assembly resolution 1514 (XV) and decision of the UK and other colonial powers not to vote against it; and, finally, to agreement on the content of the 1970 Declaration on Friendly Relations, which confirmed the existence of the right of peoples to self-determination.^132^

### D. The Implications for Stability and Change

The logic of the A-series tells us that we resolve the ‘intertemporal problem’, the problem of deciding which iteration of the law to apply, by recognising that the International Court of Justice—or any other law applier—determines the applicable law from the privileged position of ‘now’, ie with the benefit of hindsight. This does not mean the ICJ can apply any rule it chooses to past facts,^133^ and the Court has confirmed the prohibition on the retrospective application of laws.^134^ But it does mean that the ICJ’s understanding of the applicable law on a given day can change over time.

When customary international law changes, we can identify three distinct periods of time: (1) the period when there is consensus that the old rule must be applied; (2) a period of transition, when some state practice and *opinio juris* will support the old rule and some the new; and (3) the time when there is agreement that the new rule is to be applied. There must be the period of transition, because the ICJ has rejected the possibility of ‘instant custom’,^135^ the idea that a single act, such as the adoption of a General Assembly resolution, can create custom without any time passing.^136^

During the period of transition from the old customary norm (CIL N1) to the new rule (CIL N2), the International Court of Justice must apply the old rule (CIL N1) to any action that occurs during the period. This is because the period of transition is characterised, *at the time*, by three features: (i) there must be some evidence that the law is changing; (ii) there is, however, insufficient evidence to show that the new rule has crystallised; and (iii) there is the possibility that the law will not change, ie that the new rule will not crystallise. The ICJ explains the point this way: ‘The possibility of the law changing is ever present’, but this does not mean that ‘the Court should declare the law between the Parties as it might be’. The job of the Court is to ‘render a judgment on the basis of the law as it exists at the time of its decision’.^137^

Once there is agreement that the law has changed, the ICJ can note the consolidation of evidence of state practice and *opinio juris* over time to identify the moment, in the period of transition, when the law changed. This is because the same transition period is now characterised by three different features: (i) we know that the evolving patterns of state practice and *opinio juris* were evidence of the crystallisation of the new rule; (ii) we know that the new rule did crystallise; and (iii) we know that there was no return to the *status quo ante* represented by the old rule. In other words, the ICj is able to determine the moment of transition from the old rule to the new, in the certain knowledge that the law did change. There must be a moment of change, because the notion of change tells us that the old customary rule (CIL N1) cannot say something at odds with the new rule (CIL N2) at the same moment. It cannot be the case, for example, that ‘there is no right of peoples to self-determination’ (CIL N1) and that—at the same time—‘there is a right to self-determination’ (CIL N2).

Once there is agreement that the law has changed, the International Court of Justice must apply the new rule to any acts occurring after the moment of crystallisation. This will include the time in the transition period between the moment of crystallisation and the time when there is a consensus that the law has changed. This means that, with the benefit of hindsight, we can identify the following distinct periods of time when there has been a change in customary international law: (1) the period when there is agreement that the old customary norm is to be applied; (2a) the period when the new rule was crystallising, but had not yet crystallised, when the old rule must still be applied; (2b) the period after the moment of crystallisation, but before the agreement on the existence of a new general practice accepted as law, when the new rule must now be applied; and (3) the period when there is consensus that the new rule is to be applied. The point is significant because, *during the period of transition*, the ICJ must apply the old rule to any act occurring in the period of transition, including the period after the crystallisation of the new rule, but before its general acceptance (ie during period 2b). But *after there is consensus that a new rule has crystallised*, the ICJ must apply the new rule to any act occurring after the moment of crystallisation, including in the same period (2b). This means that the International Court of Justice can give a different answer as to the applicable law, depending on *when* it examines the issue.

We see this with *Chagos Archipelago*. To make sense of the Opinion on this case, we have to remember that the detachment of the Chagos Islands, in 1965, and the granting of Mauritian independence, in 1968, occurred during a period of change in the law on self-determination. Before the 1950s, there was no talk of an international law right of peoples to self-determination, so any change to the administrative boundaries of non-self-governing territories would have been lawful.^138^ The International Court of Justice dates the start of the transition period to the adoption of General Assembly resolution 637 (VII) on 16 December 1952.^139^ The transition period ended in 1970, with the adoption of the Friendly Relations Declaration, which ‘confirmed’ the customary status of the self-determination norm.^140^ During the period of transition from the old rule (‘there is no right of peoples to self-determination’) to the new (‘there is a right to self-determination’), ie in the period 1952–70, the old rule must be applied, because there is always the possibility that the self-determination norm will not crystallise. Had, then, the ICJ examined the issue in the late 1960s, it would have concluded that there is no right of peoples to self-determination, and the detachment of the Chagos Archipelago is lawful *at this time*. But, from the privileged position of 2019, the Court was able to determine that the self-determination norm crystallised with the adoption of General Assembly resolution 1514 (XV) on 14 December 1960,^141^ in the certain knowledge that the norm had crystallised. Now, the ICJ must apply the new rule to all actions from the moment of crystallisation, including those in the transition period (ie between 1960 and 1970), with the consequence that it must find that the detachment of the Chagos Archipelago was unlawful *at that time*. The only difference in these two positions is the moment when the evidence for the existence of the self-determination norm is considered, meaning that we have to take into account the passage of time, along with state practice and *opinio juris*, in the identification of customary norms.

## 7. Conclusion: The Doctrine of Intertemporal Law Explained

The objective of this article was to make sense of the doctrine of intertemporal law by explaining the importance of time in the identification of the customary rule applicable to the facts. The work first outlined the distinction, well known to philosophers of time, between McTaggart’s A-series and B-series conceptions of time, showing the importance of A- and B-series logic in the debate on the identification of custom. There is no difficulty with international lawyers relying on B-series thinking to explain the timeline of juridical events, which can be arranged in terms of being ‘earlier than’ or ‘later than’ each other. The trouble comes when they look to the tenseless logic of the B-series to determine which iteration of the rule is ‘simultaneous with’ the facts, with the requirement that any conclusion as to the legality or validity of an act must remain true for all time. But we have seen that reliance on the B-series is misplaced, because international lawyers believe that the passage of time is real and they think in terms of custom evolving through time—from the future, through the present and on to the past—as the result of contingent decisions taken by states and other international law actors. We must, then, look to the logic of the A-series conception of time—and work out the implications of this for our understanding of the doctrine of intertemporal law.

The intertemporal problem is the difficulty of determining which customary norm to apply when the law has changed. The answer is always the law contemporary with the facts. So, on the given day, we can ask, ‘What is the applicable law *at this time*?’ But the logic of the A-series tells us that we make judgments from the privileged position of ‘now’ and that tensed propositions matter. After the fact, we must always ask, ‘What was the law applicable *at that time*?’ This means that we determine the applicable law with the benefit of hindsight and that our conclusions can change over time. In other words, the central insight of A-series thinking is that, in the determination of the law applicable to the facts, it matters *when* we decide on the applicable law.

We are now in a position to outline the doctrine of intertemporal law—the rule that tells us which law to apply to the facts.

The first branch of the doctrine is well established. It requires, in the words of the *Island of Palmas* case, that ‘a juridical fact must be appreciated in the light of the law contemporary with it’.^142^ The legality or validity of an act is, then, to be judged by the standards in force at the time the act occurs. This point was confirmed by the International Court of Justice in *Chagos Archipelago*.^143^

The second branch requires that we take into account any change in the law over time. There are two parts to the second branch of intertemporal law.

The first part of the second branch confirms that new rules can impose conditions for the continued enjoyment of rights. The intertemporal doctrine does not prevent new customary rules from crystallising, with implications for the established legal positions of states. This was first explained in 1933 by Hersch Lauterpacht in response to Max Huber’s formulation of the intertemporal rule: ‘In certain cases rights may cease to be effective as the result of the development of new rules of law attaching conditions of the continued validity of these rights.’^144^ Where the law changes over time to make new demands for the continued enjoyment of existing rights, states must comply with the new requirements or risk the loss of rights already established. We see this with the impact of the self-determination norm on titles to territories obtained during the period of European colonisation. Following the crystallisation of the right of peoples to self-determination, valid title could only be maintained where the population agreed to the continuing exercise of sovereign power, and any change in the administrative boundaries of the colonised territory was only valid with the free and genuine consent of the people concerned—again, a point confirmed in *Chagos Archipelago*.^145^

The second part of the second branch of the doctrine of intertemporal law—stated here for the first time, but in line with the ICJ’s reasoning in *Chagos Archipelago*—confirms that the rights and responsibilities of states can change in time, as our understanding of the applicable law changes over time. When customary international law changes, we have to decide whether to apply the old rule or the new. Often this is not controversial, with a consensus on the period of time when the old rule is to be applied and when the new rule reflects the applicable law.^146^ During the transition period from the old rule to the new, when there is some evidence that the law is changing, but not sufficient evidence to show that the law has changed, the old rule must be applied, because there is no guarantee that the law will change. Once the law has changed, however, we must apply the new rule from the moment of its crystallisation. This will include the period between the moment of crystallisation (identified with the benefit of hindsight) and the period when there is general agreement that the new rule is to be applied, even though, at the time, we would have applied the old rule. In other words, the second branch explains that our conclusions about the applicable law on a given day, during a period of transition in the law, can change, depending on *when* we examine the available evidence of state practice and *opinio juris*.

## Footnotes

1 TO Elias, ‘The Doctrine of Intertemporal Law’ (1980) 74 AJIL 285, 293 (‘in order to apply the doctrine of intertemporal law to a given case, it is sufficient to show that the old rule has been changed to the new’).

2 George Rodrigo Bandeira Galindo, ‘Progressing in International Law’ (2010) 11 Melbourne Journal of International Law Melb. J. Int'l L. 515, 526 (time is ‘hardly discussed, with few exceptions, in recent [international law] scholarship’).

3 See Panos Merkouris, ‘(Inter)Temporal Considerations in the Interpretative Process of the VCLT: Do Treaties Endure, Perdure or Exdure?’ (2014) 45 NYIL 121, 128; Sondre Torp Helmersen, ‘Evolutive Treaty Interpretation: Legality, Semantics and Distinctions’ (2013) 6 European Journal of Legal Studies 161, 185.

4 Institut de Droit International, Resolution on ‘The Intertemporal Problem in Public International Law’, Session of Wiesbaden (1975) preamble <http://justitiaetpace.org/resolutions.php> accessed 18 September 2020.

5 Declaration of Judge Bennouna, *Sovereignty over Pedra Branca/Pulau Batu Puteh, Middle Rocks and South Ledge*, ICJ Rep 2008, 12, 128, para 2.

6 Hugh Thirlway, ‘Law and Procedure of the International Court of Justice 1960–1989: Part One’ (1989) 60 BYIL 1, 130. (‘[T]he intertemporal law principle is only adjective law – it is a technique for applying the appropriate law to the facts, not itself a rule of substantive law.’)

7 Rosalyn Higgins, ‘Time and the Law: International Perspectives on an Old Problem’ (1997) 46 ICLQ 501, 515 (few arbitral dicta ‘have been more widely cited, or have come to assume a more important place in international law’).

8 Merkouris (n 3) 128.

9 Separate Opinion of Judge Al-Khasawneh, *Land and Maritime Boundary between Cameroon and Nigeria*, ICJ Rep 2002, 303, 492, para 11.

10 Daniel-Erasmus Khan, ‘Max Huber as Arbitrator: The Palmas (Miangas) Case and Other Arbitrations’ (2007) 18 EJIL 145, 167.

11 *Island of Palmas (Netherlands v USA)* (1928) 2 RIAA 829, 845.

12 Huber is clearly sceptical about the strength of the argument, expressing ‘every reservation as to the soundness of such view’: ibid. But it is this acceptance of the validity of Spanish title in the 16th century that necessitates his consideration of the intertemporal rule, given the subsequent change in the law.

13 ibid 846 (emphasis added).

14 ibid 845.

15 We see this in Philip Jessup’s reaction to the *Island of Palmas* Award, where he expresses concern that the legal standards of today could be applied to the past actions of states, and that ‘Such a retroactive effect of law would be highly disturbing’: Philip C Jessup, ‘The Palmas Island Arbitration’ (1928) 22 AJIL 735, 740.

16 Few scholars dispute the existence of the second branch, but cf Anthony D’Amato, ‘International Law, Intertemporal Problems’ in Rudolf Bernhardt and Peter Macalister-Smith (eds), *Encyclopaedia of Public International Law* (North Holland 1992) 12344, 1235 (‘Huber’s “extension” of the doctrine of intertemporal law… has not been generally accepted’) <https://ssrn.com/abstract=2443129> accessed 18 September 2020. Whilst there are no references for the intertemporal doctrine in *Island of Palmas*, Eirik Bjorge argues that ‘both limbs of the rule of intertemporal law were well settled in intertemporal law when Huber restated them in Island of Palmas’: Eirik Bjorge, *The Evolutionary Interpretation of Treaties* (OUP 2014) 145.

17 H Lauterpacht, *The Function of Law in the International Community* (first published 1933, OUP 2011) 292.

18 Higgins (n 7) 516 (‘The Huber dictum [provides that] a State must continue to maintain a title, validly won, in an effective manner—no more and no less’).

19 Jessup (n 15) 740.

20 *Island of Palmas* (n 11) 845.

21 cf, however, ibid 870 (‘Such inchoate title, based on display of State authority, would, in the opinion of the Arbitrator, prevail over an inchoate title derived from discovery’).

22 Khan (n 10) 169.

23 For an overview of the literature, see Zhenni Li, ‘International Intertemporal Law’ (2018) 48 Cal W Int’l LJ 341.

24 James Crawford, *Brownlie’s Principles of Public International Law* (9th edn, OUP 2019) 206.

25 Malcolm Shaw, *International Law* (8th edn, CUP 2017) 378.

26 Ulf Linderfalk, ‘The Application of International Legal Norms Over Time: The Second Branch of Intertemporal Law’ (2011) 58 NILR 147, 150.

27 Institut de Droit International (n 4).

28 The work does not, therefore, deal with the application of the intertemporal rule to treaties, or the related issue of evolutionary interpretation.

29 *Legal Consequences of the Separation of the Chagos Archipelago from Mauritius in 1965*, Opinion, 25 February 2019, para 149, with reference to *North Sea Continental Shelf*, Judgment, ICJ Rep 1969, 44, para 77.

30 See General Assembly resolution 73/203, ‘Identification of Customary International Law’, adopted 20 December 2018 without a vote. See, generally, ‘Analytical Guide to the Work of the International Law Commission on “Identification of Customary International Law”’ <http://legal.un.org/ilc/guide/1_13.shtml> accessed 18 September 2020.

31 Report of the International Law Commission Sixty-third session (26 April–3 June and 4 July–12 August 2011), General Assembly Official Records Sixty-sixth Session Supplement No 10 (A/66/10), Annex A Formation and evidence of customary international law, 305 para 8(vi).

32 Michael Wood, Special Rapporteur, ‘Fourth Report on Identification of Customary International Law’, International Law Commission, Sixty-eighth Session, Geneva, 2 May–10 June and 4 July–12 August 2016 UN Doc A/CN.4/695, para 16.

33 See Separate Opinion of Judge Sepúlveda-Amor, *Dispute regarding Navigational and Related Rights*, ICJ Rep 2009, 213, 273, para 25; GI Tunkin, ‘Remarks on the Juridical Nature of Customary Norms of International Law’ (1961) 49 CLR 419, 419 (‘Duration, or in other words, the element of time, also plays an important role in the process of formation of a customary norm of international law’).

34 Draft Conclusion 8(2) (n 30).

35 ‘Draft Conclusions on Identification of Customary International Law, with Commentaries’ in Report of the International Law Commission, Seventieth session, UN Doc A/73/10 (2018), Commentary on draft Conclusion 8, para. 9. The other references to time can be found in draft Conclusion 10(3), ‘Failure to react over [a reasonable] time to a practice may serve as evidence of acceptance as law’; draft Conclusion 11(1)(a), ‘A rule set forth in a treaty may reflect a rule of customary international law if it is established that the treaty rule codified a rule of customary international law existing at the time when the treaty was concluded’. In relation to the persistent objector rule, the Commentary makes the point that ‘The timeliness of the objection is critical’: Commentary on draft Conclusion 15, para. 5.

36 *North Sea Continental Shelf* cases, ICJ Rep 1969, 3, para 74.

37 Dissenting Opinion of Judge Tanaka, ibid 172, 178.

38 International Law Association, Final Report of the Committee on Formation of Customary (General) International Law, ‘Statement of Principles Applicable to the Formation of General Customary International Law’, Conference Report London (2000) 20 <www.ila-hq.org/index.php/committees> accessed 18 September 2020.

39 Gerald Postema makes the point that most international lawyers think of custom as the addition of two factors, i.e., ‘behavior or usage (*usus*) plus belief or conviction of (legal) necessity (*opinio juris sive necessitatis*)’: Gerald J Postema, ‘Custom in International Law: A Normative Practice’ in Amanda Perreau-Saussine and James Bernard Murphy (eds), *The Nature of Customary Law* (CUP 2007) 279.

40 *Military and Paramilitary Activities in and against Nicaragua*, ICJ Rep 1986, 14, para 207 (‘for a *new* customary rule to be formed, not only must the acts concerned “amount to a settled practice”, but they must be accompanied by the *opinio juris*’ (emphasis added)).

41 Separate Opinion of Judge Cançado Trindade, *Request for Interpretation of the Judgment of 15 June 1962 in the Case concerning the Temple of Preah Vihear*, ICJ Rep 2011, 537, 566, para 12 (reference omitted) (emphasis added).

42 The philosopher, Alexander Gunn explains that our sense that the passage of time is real and continuous is largely a consequence of things changing: J Alexander Gunn, ‘The Problem of Time’ (1929) 4(14) Journal of Philosophical Studies 180, 183. The problem is that if the only way you can speak about time is in terms of change, it must be the case that, if nothing changed, no time would pass. See PJ Zwart, ‘The Flow of Time’ (1972) 24 Synthese 133, 135.

43 For scientists, the fundamental unit of clock time is the ‘second’, with 60 seconds making 1 minute, and 60 minutes 1 hour. The second is defined as the frequency with which caesium atoms change from one state to another 9,192,631,770 times. See Jonathan D Betts, ‘Second: Unit of Time’ in *Encyclopædia Britannica* (2011) <www.britannica.com/science/second> accessed 18 September 2020. So when we say that a certain amount of time has passed, according to our clock, all we are saying is that one event (the ticking of a clock a certain number of times) had the same duration as another event (a caesium atom moving a certain number of times). We cannot say, objectively, that any time passed. See Zwart (n 42) 140.

44 HG Wells, *The Time Machine* (first published 1895, Vintage) 2.

45 Isaac Newton, *The Principia: The Authoritative Translation and Guide* (University of California Press 2016) 408 (emphasis added).

46 Klaus Mainzer, *The Little Book of Time* (Josef Eisinger tr, Springer 2002) 43.

47 Adam Frank, *About Time* (Oneworld 2011) 134.

48 Ori Belkind, *Physical Systems: Conceptual Pathways between Flat Space-time and Matter* (Springer 2012) 103.

49 JJC Smart, ‘The River of Time’ (1949) 58(232) Mind 483, 483.

50 Zwart (n 42) 135.

51 J. Ellis McTaggart, ‘The Unreality of Time’ (1908) 17 Mind 457, 458.

52 ibid 461.

53 ibid 468 (emphasis added).

54 Heather Dyke, ‘Time and Tense’ in Adrian Bardon and Heather Dyke (eds), *A Companion to the Philosophy of Time* (Wiley Blackwell 2013) 328, 335.

55 CD Broad, *Scientific Thought* (Kegan Paul 1923) 54.

56 Ryan Wasserman, ‘The Problem of Change’ (2006) 1(1) Philosophy Compass 48, 50.

57 Dean W Zimmerman, ‘The A-Theory of Time, The B-Theory of Time, and “Taking Tense Seriously”’ (2005) 59 Dialectica 401, 405.

58 Adrian Bardon, *A Brief History of the Philosophy of Time* (OUP 2013) 83.

59 MM Schuster, ‘Is the Flow of Time Subjective?’ (1986) 39 Review of Metaphysics 695, 697.

60 See M Joshua Mozersky, ‘The B-Theory in the Twentieth Century’ in Bardon and Dyke (n 54) 167, 173–4.

61 John W Carroll and Ned Markosian, *An Introduction to Metaphysics* (CUP 2010) 164 (emphasis in original).

62 The International Court of Justice has confirmed the possibility of change in customary international law. See eg *Military and Paramilitary Activities in and against Nicaragua*, ICJ Rep 1986, 14, para 207 (reference to ‘a modification of customary international law’); *Ahmadou Sadio Diallo*, ICJ Rep 2007, 582, 615 (‘a change in the customary rules’).

63 Michael Byers, *Custom, Power, and the Power of Rules: International Relations and Customary International Law* (CUP 1999) 158.

64 Sir Gerald Fitzmaurice, ‘The General Principles of International Law: Considered from the Standpoint of the Rule of Law’ (1957) 92 RdC 1, 113. See also GJH Hoof, *Rethinking the Sources of International Law* (Kluwer 1983) 99 (‘It must be quite an extraordinary system of law which incorporates as its main, if not the only, vehicle for change the violation of its own provisions’).

65 *North Sea Continental Shelf*, Judgment, ICJ Rep 1969, 44, para 77 (‘evidence of a belief that this practice is rendered obligatory *by the existence* of a rule of law requiring it’ (emphasis added)).

66 Byers (n 63) 131 (emphasis in original).

67 Hugh Thirlway, *The Sources of International Law* (OUP 2014) 81.

68 James Crawford (with Thomas Viles), ‘International Law on a Given Day’ in James Crawford, *International Law as an Open System: Selected Essays* (Cameron May 2002) 69, 92.

69 An event can be something that happens at a certain moment or something that endures for a certain period: Gunn (n 42) 186. Gunn gives the examples of the death of Marat and the French Revolution.

70 In *North Sea Continental Shelf*, ICJ Rep 1969, 3, the International Court of Justice notes that the Truman Proclamation ‘soon came to be regarded as the starting point of the positive law on the subject, and the chief doctrine it enunciated … came to prevail over all others’ (para 47). The judgment then talks about ‘the legal régime established *subsequent to* the Truman Proclamation’ (para 97 (emphasis added)).

71 The chronological paradox is reflected in the fact that the United States cannot genuinely have believed that the new rule was ‘already’ accepted as law when it made the Proclamation.

72 The question of international law on a given day is influenced by Joseph Raz’s idea of a ‘momentary legal system’, which is distinguished from a ‘legal system’. A momentary legal system contains all the laws of a system ‘valid at a certain moment’. Thus, an English law enacted in 1906 and repealed in 1927 and an English law enacted in 1948 belong to the same legal system, but ‘there is no momentary legal system to which both belong, because they were never valid at one and the same moment: Joseph Raz, *The Concept of a Legal System* (2nd edn, OUP 1980) 34.

73 James Crawford, *Chance, Order, Change: The Course of International Law* (Hague Academy of International Law 2014) 81–2.

74 ibid.

75 ibid, with reference to JR Crawford, ‘Keynote Speech: The Identification and Development of Customary International Law’ (Spring Conference of the ILA British Branch, 23 May 2014).

76 ibid (emphasis added). Wood quotes Karol Wolfke, *Custom in Present International Law* (2nd rev edn, Martinus Nijhoff 1993) 54 (International lawyers ‘are, in general, in agreement that the moment of formation of a custom … cannot be ascertained, since it is practically speaking intangible’).

77 ibid.

78 There is no UK equivalent to the Truman Proclamation. 1964 is the year that the UK ratifies the Geneva Convention on the Continental Shelf and adopts the Continental Shelf Act. See, generally, Geoffrey Marston, ‘Incorporation of Continental Shelf Rights into United Kingdom Law’ (1996) 45 ICLQ 13.

79 JL Brierly, *The Law of Nations: An Introduction to the International Law of Peace* (6th (rev) edn, Clarendon Press 1963) 213–14.

80 Lord McNair, for example, comments, in 1963, that ‘the continental shelf is such a new concept that it is difficult to say that there is any international law upon it’: Second Reading Debate on the Continental Shelf Bill in the House of Lords, HL Deb 3 December 1963, vol 253, cols 924–5, quoted by Marston (n 78) 47.

81 Crawford, *Brownlie’s Principles* (n 24) 256.

82 See Thirlway, ‘Law and Procedure’ (n 6) 133 (‘it may also be said of an event which is to occur in the future that the law applicable to it will be the law as it stands then’).

83 ibid (‘Thus a Court is tacitly applying the intertemporal principle in every ordinary case by determining the dispute by reference to the law as it stands’).

84 *Western Sahara*, Advisory Opinion, ICJ Rep 1975, 12, para 79.

85 See eg *Minquiers and Ecrelzos*, ICJ Rep 1953, 47, 56; *Right of Passage over Indian Territory*, ICJ Rep 1960, 6, 37, 43–4; *Dispute regarding Navigational and Related Rights*, ICJ Rep 2009, 213, para 47; *Jurisdictional Immunities of the State*, ICJ Rep 2012, 99, paras 58 and 93.

86 See *Legal Consequences of the Separation of the Chagos Archipelago from Mauritius in 1965*, Opinion, 25 February 2019 (hereinafter *Chagos Archipelago*) para 148.

87 *Legal Consequences of the Separation of the Chagos Archipelago from Mauritius in 1965*, Written Statement by the United Kingdom, 15 February 2018, para 8.77 (emphasis added).

88 ibid para 8.6.

89 C Don Johnson, ‘Toward Self-Determination: A Reappraisal as Reflected in the Declaration on Friendly Relations’ (1973) 3 Ga J Int’l & Comp L 145, 158.

90 *South West Africa*, Second Phase, Judgment [1966] ICJ Rep 6. Even the minority judgments in the case do not say anything about the status of the right of peoples to self-determination.

91 Rosalyn Higgins, *The Development of International Law through the Political Organs of the United Nations* (OUP 1963) 100 (self-determination is regarded ‘as a legal right enforceable here and now’). She continues: ‘It therefore seems inescapable that self-determination has developed into an international legal right’, although ‘The extent and scope of the right is still open to some debate’ (103).

92 Writing in 1994, Higgins notes the ‘differing early views on the status of self-determination as a legal norm’, including her own 1963 contribution: Rosalyn Higgins, *Problems and Process: International Law and How We Use It* (OUP 1994) 113.

93 *Chagos Archipelago* (n 86) para 150.

94 ibid para 152.

95 ibid para 155.

96 ibid para 177 (‘The Court having found that the decolonization of Mauritius was not conducted in a manner consistent with the right of peoples to self-determination, it follows that the United Kingdom’s continued administration of the Chagos Archipelago constitutes a wrongful act entailing the international responsibility of that State … It is an unlawful act of a continuing character which arose as a result of the separation of the Chagos Archipelago from Mauritius’).

97 *Legal Consequences of the Separation of the Chagos Archipelago from Mauritius in 1965*, Written Statement by the Republic of Cyprus, 11 May 2018, para 9.

98 See Written Statement by the United Kingdom (n 87) para 8.6 (‘the International Court of Justice should apply the customary law as it stood [in the period 1965 to 1968]’); *Chagos Archipelago* (n 86) para 140 (the key events were the detachment of the Chagos Archipelago in 1965 and the independence of Mauritius in 1968: ‘It is therefore by reference to this period that the Court is required to identify the rules of international law that are applicable to that process’).

99 See Response of the United Kingdom to Judge Cancado Trindade, para 3(b): ‘The negotiating records and explanations of vote reveal that there were divided views to [the significance of General Assembly resolution 1514 (XV)], which … did not reflect States’ acceptance of a customary obligation at that time.’

100 Written Statement by the United Kingdom (n 87), para 8.75.

101 ibid para 8.77.

102 *Chagos Archipelago* (n 86) para 139.

103 ibid para 142.

104 ibid para 143 (emphasis added).

105 ibid para 155.

106 On this point, see *Jurisdictional Immunities of the State* (n 85) paras 58 and 93.

107 House of Commons Hansard Written Statement, 30 April 2109, HCWS1528 (British Indian Ocean Territory). See, generally, Jon Lunn, *Disputes over the British Indian Ocean Territory* (House of Commons Library 2019).

108 Manley O Hudson, ‘The Effect of Advisory Opinions of the World Court’ (1948) 42 AJIL 630, 630.

109 *Interpretation of Peace Treaties*, Advisory Opinion, ICJ Rep 1950, 65, 71. The function of an Advisory Opinion is to advise the UN organ that requested it: Charter of the United Nations, art 96. To do this, the Court must determine the applicable law; by doing so, it can sometimes give an ‘opinion’ on the legal position of a particular state (see *Chagos Archipelago* (n 86) para 137). But advisory opinions are not binding in the same way as contentious judgments.

110 Edvard Hambro, ‘The Authority of the Advisory Opinions of the International Court of Justice’ (1954) 3 ICLQ 2, 6. The ICJ has made clear that there is no difference in the way it reaches its conclusions in contentious and advisory cases: *Judgements of the Administrative Tribunal of the ILO upon complaints made against the UNESCO*, Advisory Opinion, ICJ Rep 1956, 77, 84.

111 *Certain expenses of the United Nations*, Advisory Opinion, ICJ Rep 1962, 151, 158. Hugh Thirlway makes the point that, if the ICJ ‘advises’ that a state is responsible for a violation of international law, that state ‘will be in a weak position if it seeks to argue that the considered opinion of the Court does not represent a correct view of the law’: HWA Thirlway, *The International Court of Justice* (OUP 2016) 139.

112 Dissenting Opinion of Sir Percy Spender, *Temple of Preah Vihear*, ICJ Rep 1962, 6, 101, 128. See also Gerald Fitzmaurice, ‘The Law and Procedure of the International Court of Justice, 1951–54: General Principles and Sources of Law’ (1953) 30 BYIL 1, 5 (‘[I]t is not permissible to import into the legal evaluation of a previously existing situation[,] doctrines of modern law that did not exist or were not accepted at the time, and only resulted from the subsequent development or evolution of international law’).

113 Einstein said on the occasion of the death of his colleague, Michele Angelo Besso: ‘People like us, who believe in physics, know that the distinction between past, present and future is only a stubbornly persistent illusion’: quoted by Bardon (n 58) 96–7.

114 Roger Teichmann draws a useful analogy between our acceptance of the passage of time and our acceptance of the reality of colours, pointing out that we accept the reality of colours, even though colours do not exist as an objective feature of the physical universe—they are simply a way that our brain interprets reflected light waves: Roger Teichmann, ‘Time and Change’ (1993) 43 Philosophical Quarterly 158, 165.

115 On this point, see John R Searle, *Making the Social World* (OUP 2010) 7.

116 Thomas Schultz, ‘Life Cycles of International Law as a Noetic Unity: The Various Times of Law-Thinking’ (2017) King’s College London Law School Research Paper No 2017-09, 6 <https://ssrn.com/abstract=2890583> accessed 18 September 2020.

117 On this point, see Rebecca R French, ‘Time in the Law’ (2001) 72 U Colo L Rev 663, 668.

118 See Hugh Thirlway, ‘Reflections on Lex Ferenda’ (2001) XXXII NYIL 3, 9 (‘a possible rule passes through a chrysalis stage of being merely ferenda, before emerging … into full glory as a rule of positive law’); Michel Virally, ‘To What Extent Are the Traditional Categories of Lex Lata and Lex Ferenda Still Viable?’ in Antonio Cassese and Joseph HH Weiler (eds), *Change and Stability in International Law-Making* (De Gruyter 1988) 66, 72 (‘lex ferenda is a law which is not yet established, which has not yet become lex lata, is in the process of becoming so’).

119 See Martti Koskenniemi, ‘Law, Teleology and International Relations: An Essay in Counterdisciplinarity’ (2012) 26 International Relations 3, 23 (international law ‘looks both backwards and forwards in time’); Philip Allott, ‘International Law and the Idea of History’ (1999) 1 Journal of the History of International Law 1, 1 (international law is ‘a bridge between the social past and the social future through the social present’).

120 See The Nobel Prize, ‘Ilya Prigogine’ <www.nobelprize.org/prizes/chemistry/1977/prigogine/facts/> accessed 18 September 2020.

121 Ilya Prigogine, *From Being to Becoming: Time and Complexity in the Physical Sciences* (WH Freeman 1980) 13.

122 On customary international law as a complex system, see Steven Wheatley, *The Idea of International Human Rights Law* (OUP 2019) ch 5.

123 See Klaus Mainzer, *The Little Book of Time* (Springer 2002) 103–5. In classical physics, by way of contrast, most phenomena are time reversible, ie the mathematics of a physical process work in either temporal direction. See Barry Dainton, *Time and Space* (Acumen 2001) 439.

124 For a good introduction to complexity, see Melanie Mitchell, *Complexity: A Guided Tour* (OUP 2009).

125 See ILC, Draft Conclusion 6(1) on Identification of customary international law (n 30).

126 Sir Hersch Lauterpacht describes this in terms of ‘the complexities—indeed the mysteries—of the rise of binding customary law from amidst the amorphous and, when taken in isolation, inconclusive manifestations of conduct’: Hersch Lauterpacht, *The Development of International Law by the International Court* (first published 1958, Grotius 1982) 390.

127 Krzysztof Skubiszewski, ‘Elements of Custom and the Hague Court’ (1971) 31 Zeitschrift für Auslandisches Öffentliches Recht und Völkerrecht 810, 812.

128 Laurence R Helfer and Ingrid B Wuerth, ‘Customary International Law: An Instrument Choice Perspective’ (2016) 37 Mich J Int’l Law 563, 575.

129 James Crawford describes this in terms of states being in a dialogical relationship with international law, ‘shaping it and shaped by, making it and made by it’: James Crawford, *Chance, Order, Change: The Course of International Law* (Hague Academy of International Law 2014) 19.

130 Prigogine (n 121) 106.

131 Ilya Prigogine and Isabelle Stengers, *Order Out of Chaos: Man’s New Dialogue with Nature* (Flamingo 1985) 170, 176. Prigogine later applied the insight to human societies: I Prigogine, ‘The Networked Society’ (2000) VI(1) Journal of World-Systems Research 892.

132 See, generally, Steven Wheatley, *Democracy, Minorities and International Law* (CUP 2005) ch 2.

133 cf Diego Mejía-Lemos, ‘The “Quimbaya Treasure,” Judgment SU-649/17’ (2019) 113 AJIL 122.

134 On this point, see *Jurisdictional Immunities of the State* (n 85) paras 58 and 93.

135 The notion of ‘instant custom’ was first proposed by Bing Cheng in the 1960s, in the context of the rapid development of a body of space law: Bin Cheng, ‘United Nations Resolutions on Outer Space: “Instant” International Customary Law?’ (1965) 5 Indian Journal of International Law 23.

136 *North Sea Continental Shelf* cases, ICJ Rep 1969, 3, para 74.

137 *Fisheries Jurisdiction*, ICJ Rep 1974, 3 para 40.

138 art 1(2) of the Charter of the United Nations establishes that the purposes of the organisation include the development of friendly relations, ‘based on respect for the *principle* of equal rights and self-determination of peoples’ (emphasis added). There is no *right* of peoples to self-determination in the Charter, although the ‘principle’ underpins ch XI (Non-Self-Governing Territories) and ch XII (International Trusteeship System). See Wheatley (n 132) 68–9.

139 *Chagos Archipelago* (n 86) para 150 (‘Prior to [resolution 1514 (XV)], the General Assembly had affirmed on several occasions the right to self-determination (resolutions 637 (VII) of 16 December 1952, 738 (VIII) of 28 November 1953 and 1188 (XII) of 11 December 1957) and a number of non-self-governing territories had acceded to independence’).

140 ibid para 155.

141 ibid para 150.

142 *Island of Palmas* (n 11) 845.

143 *Chagos Archipelago* (n 86) para 140.

144 Lauterpacht, *The Function of Law* (n 17) 291.

145 *Chagos Archipelago* (n 86) para 172.

146 Maurice Mendelson, ‘The Subjective Element in Customary International Law’ (1995) 66 BYIL 177, 203.

## Notes

My thanks to Russell Buchan, Jean D’Aspremont and Valentina Vadi for their comments on an earlier version. Thanks also for the comments of the Journal’s anonymous reviewers. The usual caveat applies.

